# The Mothers, Infants and Lactation Quality (MILQ) Study: Introduction and Study Design

**DOI:** 10.1016/j.advnut.2025.100499

**Published:** 2025-10-28

**Authors:** Lindsay H Allen, Sophie E Moore, Gilberto Kac, Kim F Michaelsen, Christian Mølgaard, M Munirul Islam, Setareh Shahab-Ferdows, Sophie Hilario Christensen, Jack I Lewis, Janet M Peerson, Xiuping Tan, Daphna K Dror, Andrew M Doel, Maria Andersson, Daniela de Barros Mucci, Amanda C Figueiredo, Bruna C Schneider, Farhana Khanam, Adriana Divina de Souza Campos, Gabriela Torres Silva, Fanta Nije, Mehedi Hasan, Anura V Kurpad, Sarita Devi, Kerry S Jones, Daniela Hampel

**Affiliations:** 1 US Department of Agriculture, ARS Western Human Nutrition Research Center, University of California Davis, Davis, California, USA; 2 Department of Nutrition, Institute for Global Nutrition, University of California, Davis, California, USA; 3 Medical Research Council Unit The Gambia at London School of Hygiene & Tropical Medicine, Fajara, The Gambia, West Africa; 4 Department of Women and Children’s Health, King’s College London, London, United Kingdom; 5 Nutritional Epidemiology Observatory, Josué de Castro Nutrition Institute, Federal University of Rio de Janeiro, Rio de Janeiro, RJ, Brazil; 6 Department of Nutrition, Exercise and Sports, Faculty of Science, University of Copenhagen, Copenhagen, Denmark; 7 Nutrition Research Division, International Centre for Diarrhoeal Disease Research, Bangladesh, Dhaka, Bangladesh; 8 Center for Clinical Research and Prevention, Copenhagen University Hospital - Bispebjerg and Frederiksberg Hospital, Copenhagen, Denmark; 9 Nutrition Research Unit, Children’s Research Centre, University Children’s Hospital Zurich - Eleonore Foundation, Zurich, Switzerland; 10 Department of Basic and Experimental Nutrition, Rio de Janeiro State University, Rio de Janeiro, Brazil; 11 Department of Physiology, St. John’s Medical College, St. John's National Academy of Health Sciences, Bangalore, India; 12 Division of Nutrition, St. John’s Research Institute, St. John’s National Academy of Health Sciences, Bangalore, India; 13 Nutritional Biomarker Laboratory, Medical Research Center Epidemiology Unit, University of Cambridge, Cambridge, United Kingdom

**Keywords:** human milk composition, human milk, nutrient concentrations, milk volume, reference values for nutrients, lactation, infant nutrition

## Abstract

The World Health Organization recommends exclusive breastfeeding for the first 6 mo of life and continued breastfeeding for 2 y or beyond. However, limited reliable, representative data on nutrient concentrations in milk from well-nourished mothers are available. Furthermore, there is a lack of data integrating human milk nutrient concentrations with the volume of milk transferred to infants during progressive stages of lactation. Accurate quantification of nutrient concentrations and milk volume is essential for setting macro- and micronutrient intake recommendations for infants and women’s additional requirements for lactation. This first article in a series of 7 in this Supplement describes the Mothers, Infants, and Lactation Quality (MILQ) study conducted at sites in Bangladesh, Brazil, Denmark, and The Gambia. The MILQ study measured human milk nutrient concentrations and quantified milk volume throughout the first 8.5 mo of lactation. Validated analytical methodologies were used for nutrient quantification. The stable isotope dilution dose-to-mother method was used for milk volume measurement. A total of 1242 mother–infant dyads participated in the MILQ study. Milk volumes, milk nutrient concentrations, percentile curves, and total nutrient intakes (concentration times milk volume at each time point) are presented in the series of articles in this supplement. Comparisons are made between values in the MILQ study and those used by the Institute of Medicine (now renamed the National Academy of Medicine) to set nutrient intake recommendations for infants and lactating women, and with other selected studies. Data from the MILQ study provide a valuable resource for updating existing nutrient intake recommendations, evaluating and improving infant nutrition strategies, and assessing interventions to optimize maternal and infant nutritional status and health.

**Statement of significance**

Published values for the concentrations of most nutrients in human milk, especially for vitamins and other micronutrients, are sparse and inconsistent, yet they are used to set recommended nutrient intakes for infants and lactating women. The MILQ study is the first to establish reference values for nutrient concentrations in human milk through 8.5 mo of lactation, accompanied by measures of milk volume, enabling the calculation of total intakes by infants, thus improving nutrient intake recommendations for infants and lactating women.

## Introduction

The WHO recommends exclusive breastfeeding for the first 6 mo of life and continued breastfeeding up to or beyond 2 y of age [1]. However, there is limited reliable data on the quality of human milk concerning many nutrients, especially micronutrients [2]. The most recent global data on milk composition, published nearly 4 decades ago, were collected in the 1985 WHO Collaborative Study on Breast-Feeding, which analyzed milk volume and limited data on the number of macro- and micronutrients in milk from a large cohort of women in Chile, Ethiopia, Guatemala, Hungary, India, Nigeria, the Philippines, Sweden, and the Democratic Republic of Congo [3]. In 2020, the National Academies Press published a report scanning for new evidence on the nutrient content of human milk to address the lack of integrated age-specific nutrient requirements [4].

Adequate nutrient transfer to infants in the first 6 mo of life is critical, with early onset wasting prevalent across multiple countries [5]. Poor maternal nutritional status and/or insufficient nutrient intake can impact the concentration of many nutrients in human milk, potentially compromising infant growth and development [6]. Reference values (RVs) for nutrient concentrations in milk from well-nourished mothers are needed for multiple purposes, including to establish recommended nutrient intakes for infants and lactating women and to assess the need for improvements in maternal micronutrient status during lactation. Additionally, these help evaluate the efficacy and effectiveness of nutritional interventions for lactating women.

In their 2018 review of the evidence used to set recommended nutrient intakes for infants and lactating women, Allen et al. [7] described the small number of participants from whom samples were collected in many studies and the variability in milk collection methods. Furthermore, the need to evaluate the validity of analytical methods for measuring nutrients in the complex milk matrix has been largely overlooked [8,9]. Thus, for many nutrients, there are few or no valid data available to estimate the amount of nutrients secreted into the milk of healthy, well-nourished women and consumed by their infants. To establish appropriate RVs for nutrient concentrations in human milk, it is necessary to collect samples using a standardized protocol at different times during lactation from well-nourished, healthy women in diverse countries; avoid samples collected from women receiving multi-micronutrient supplementation and/or fortified foods; account for any possible influence of maternal nutritional status and diet; and apply analytical methods that have been validated for accuracy and sensitivity in human milk matrix.

The main objective of the series of articles published in this Supplement is to provide global RVs for the concentrations of macro- and micronutrients in human milk collected from well-nourished women through the first 8.5 mo of lactation. The data were obtained in the multi-center Mothers, Infants, and Lactation Quality (MILQ) and Early MILQ (E-MILQ) studies, with sites in Bangladesh, Brazil, Denmark, and The Gambia. Milk volume was also measured to quantify nutrient transfer to the infant; this is more accurate than the usual assumption that every infant consumes 0.78 L of milk per day throughout the first 6 mo of lactation. The MILQ and E-MILQ studies also collected samples to analyze maternal and infant nutritional status biomarkers, human milk oligosaccharides and specific proteins, the human milk and stool microbiome, the milk and plasma metabolomes, and the maternal genome. Articles exploring such data will be published elsewhere in the future.

## Methods

### Study design

The MILQ study started in 2017, and the final milk sample was collected in 2022 (Denmark: September 2017 to December 2019, Brazil: January 2018 to March 2022, Bangladesh: April 2018 to October 2021, The Gambia: May 2018 to March 2022). Sites were chosen based on the following criteria: population of well-nourished women of childbearing age; cultural practice of exclusive and prolonged breastfeeding based on national data; minimal maternal nutrient supplementation or dietary fortification; investigators experienced in perinatal research; and geographical diversity.

**FIGURE 1:**
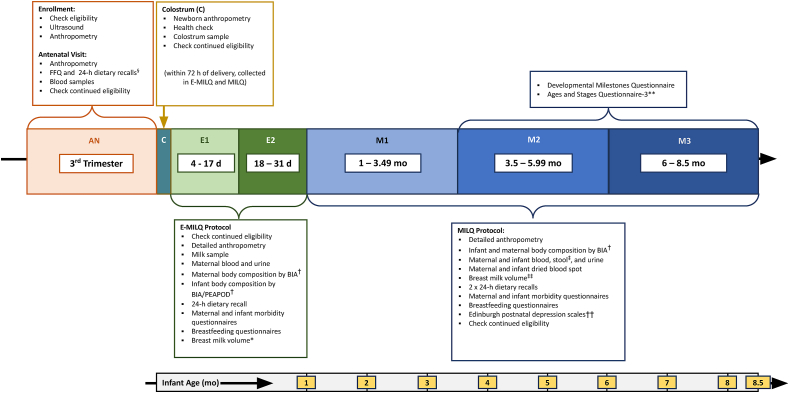
Study timeline and data collection. ∗Due to ^2^H_2_O human milk volume protocol timings (^2^H_2_O dose given to mother at baseline followed by infant saliva collected at baseline, 1, 2, 3, 4, 13, and 14 d after dosing), the protocol was performed on the mother–infant pair a single time during 4–31 d, except in Brazil, where the protocol was not performed at all during this time range. ^†^E2 only. Body composition was not measured in Bangladesh. Infant body composition was measured using Bioimpedance in Brazil and Denmark and PEA POD Air Displacement Plethysmography in The Gambia. ^§^24-h recall not collected in The Gambia. ^‡^Stool samples were not collected in Bangladesh or Denmark. ^∗∗^Only at M3 in The Gambia and Bangladesh, M1-3 in Brazil. ^††^Not collected in Denmark; also collected at E1-2 in Brazil. ^‡‡2^H_2_O except in Denmark, which used 24-h before- and after-weighing.

Sample collection was distributed across visit windows (C [0–3 d]; M1 [1–3.49 mo], M2 [3.5–5.99 mo], M3 [6–8.5 mo]) to obtain values for the entire period from 1 to 8.5 mo postpartum (Figure 1). An add-on study, E-MILQ, was designed to collect milk samples during the first month of lactation (Figure 1; C [0–3 d], E1 [4–17 d], and E2 [18–31 d]) to capture the crucial period when the concentration of some nutrients in colostrum and milk undergo rapid changes. Furthermore, the mean 14-day milk volume was measured for each participant between 4 d and 31 d postpartum to quantify nutrient transfer to the infant accurately. The E-MILQ collection window was not included in the original MILQ design due to concerns about overloading the participants in the early postpartum period. In Brazil and The Gambia, some women were enrolled in both the MILQ and E-MILQ studies due to recruitment delays resulting from the COVID-19 pandemic, but in Bangladesh and Denmark, different women provided samples in the 2 studies.

The planned sample size of 250 mother–infant dyads at each of the 4 sites in the MILQ study was calculated based on the following: *1)* estimating the fifth and 50th percentiles of the concentration of each nutrient in milk within each site, and *2)* establishing equivalence across study sites before pooling data. The calculated sample size avoids overlap between the 95% CI around the fifth and 10th centiles and estimates the sample median within 5 centiles with 95% CI [10]. The planned sample size of the E-MILQ study was 65 mothers per site (260 in total), calculated based on a 95% CI for estimating the sample median to within 5 centiles in the constructed RV curves for each nutrient.

Mothers were given context-specific, detailed study information and enrolled after providing written informed consent. Ethical approval was granted to the study coordinating center by the institutional review board (IRB) of the University of California, Davis, CA, USA (IRB ID: 920618–1, Protocol HRP-503- MILQ IRB, Department of Health and Human Services FWA No: 00004557). Each site obtained local approval: the Internal Review Boards of the International Centre for Diarrhoeal Disease Research, Bangladesh (icddr,b; PR-17085), the Brazilian National Commission for Research Ethics (Project numbers: 64767717.4.0000.5275, 40093120.4.0000.5257; protocols 2.086.708, 2.875.218, 4.865.685, 4.674.897) and the Research Ethics Committees of the Maternity School of the Federal University of Rio de Janeiro (1.948.992, 2.769.611, 4.449.007), of the University Hospital Clementino Fraga Filho of the Federal University of Rio de Janeiro (4.574.571) and of the Municipal Health Secretariat of Rio de Janeiro, Brazil (2.100.255, 3.012.400, 4.925.413), the Committees on Biomedical Research Ethics for the Capital Region of Denmark (H-17015174), and the joint Gambia Government/Medical Research Council Unit The Gambia Ethics Committee (SCC 1572v1.1, Project ID/ethics ref: 22768). The study was registered at www.isrctn.com (Ref. NCT03254329) before enrollment initiation, and the complete study protocol is published elsewhere [10].

Women were screened and recruited during antenatal visits before or during the third trimester of pregnancy. In Brazil, after the onset of COVID-19, women were screened and recruited postpartum in the maternity ward. Maternal inclusion criteria were as follows: age 18–40 y; BMI 18.5-29.9 kg/m^2^ pre-pregnancy (Brazil and Denmark) or <2 wk postpartum (Bangladesh and The Gambia); mid-upper arm circumference 23–33 cm (The Gambia) or 21–33 cm (Bangladesh); height ≥ 150 cm (>145 cm in Brazil); hemoglobin ≥100 g/L (not verified in Denmark); no relevant past or current medical problems; alcohol intake <50 mL/d in Denmark, <30 g/wk in Brazil; 0 in Bangladesh, and not a criterion in The Gambia because consumption of alcohol in this population is uncommon; non-smoking; non-vegan or macrobiotic diet; no multi-micronutrient supplementation except iron and folic acid (all sites) and calcium and vitamin D (Bangladesh and Denmark); low habitual intake of fortified foods except iodized salt; uncomplicated singleton pregnancy; and delivery at 37–42 wk of gestation. Maternal diet quality was assessed at screening using a dietary diversity questionnaire (except in Denmark); women were included if they consumed an estimated >15 g/d (one tablespoon) from ≥5 of 10 food groups (or 4 of 8 food groups in Brazil) [11]. Infant inclusion criteria at birth were birthweight 2500–4200 g and absence of congenital abnormalities interfering with feeding, growth, or development. At E1-2 and M1-3, mother–infant dyads were excluded for cessation of breastfeeding [if not exclusive breastfeeding through visit M1 with an exception from birth until 7 d (3 d in E-MILQ), and if not partially breastfeeding through visit M3]; serious maternal or infant illness; maternal consumption of micronutrient supplements other than iron and folic acid (or vitamin D and calcium in Bangladesh and Denmark); or infant undernutrition (Z-score <−2.0 for length-for-age, weight-for-age, or weight-for-length) [10].

### Sample and data collection

The MILQ study methods have been described in detail [10] and were standardized across sites through the internal “Manual of Operations and Procedures” [10]. Data and biological samples collected at the antenatal (AN) and postpartum visits included maternal anthropometry (AN, E1-2, M1-3); infant anthropometry (C, E1-2, M1-3); maternal and infant body composition (E2, M1-3); dietary intake FFQ (AN) and 24-h dietary recalls (1 each at E1-2, 2 each at AN, M1-3); colostrum (C); milk and milk volume (E1-2, M1-3; except milk volume in Brazil at E1-E2 and a subset of M1-M3 due to COVID-19 regulations); maternal blood (AN, E2, M1-3), stool (M1-3) and urine (E1-2, M1-3); and infant blood, urine, and stool (M1-3); maternal and infant dried blood spot (DBS) (M1-M3), and infant morbidity (E1-2, M1-3). Questionnaires about breastfeeding practices, socioeconomic status, postpartum depression (Edinburgh Postnatal Depression Scales [EPDS]), and infant development and milestones (Ages and Stages Questionnaire) were administered at various times during the study period (Figure 1). For cultural and practical purposes, some procedures differed by site; for example, maternal stool samples were not collected in Bangladesh or Denmark, body composition was not measured in Bangladesh, and at M1-3 in Denmark, milk volume was assessed by 24-h before- and after-feed weighing, and the EPDS questionnaire was not administered.

Milk samples were collected by full breast expression from the opposite breast last used for feeding. Each site used the Medela Symphony electric hospital-grade breast pump (Medela). Milk samples were collected between 09:00 and 14:00 into 250 mL aluminum-covered collection bottles that were stored away from direct light. After mixing full expressions and aliquoting the sample according to the study protocol [10], the remaining milk was offered back to the mother.

Each site entered data directly into a dedicated Research Electronic Data Capture (https://www.project-redcap.org/) database [12], managed by the Medical Research Council Unit, The Gambia. After being checked for permissible ranges, suspect or missing data were flagged and returned to data managers at each site for correction. In addition, each site checked for errors in its own data set. At the end of the study, all data were merged centrally at the USDA, Agricultural Research Service (ARS) Western Human Nutrition Research Center in Davis, CA. Laboratory data were added to this database as they became available. Samples and data had unique identifiers consisting of a country code, a code for mother/infant, a 3- or 4-digit dyad number, a check letter, and a visit number.

Biological samples were frozen at −80°C, except for saliva and DBS at −20°C in The Gambia and Bangladesh, before being shipped on dry ice to the Western Human Nutrition Research Center, where most nutrient analyses were performed, or to other analytical laboratories. Vitamin D analysis of milk samples took place at the Medical Research Council Epidemiology Unit, University of Cambridge, UK. Iodine concentration in milk and urine, and thyroid function parameters in DBS were measured at the University Children’s Hospital in Zurich, Switzerland.

### Analytical methods

The recent development of valid analytical methods for vitamins in human milk matrix is a major factor enabling the accumulation of new information on milk composition. In particular, the introduction and use of liquid chromatography-tandem mass spectrometry for measuring the concentrations of most B vitamins [13] and vitamin D [14] has allowed for more sensitive quantification of these nutrients. Macronutrients were analyzed using near-infrared spectrometry on a SpectraStar XT Neonatal Analyzer (Unity Scientific). Inductively coupled plasma-mass spectrometry was used to measure multiple minerals simultaneously [15]. Care was taken to validate these methods; indeed, the development and publication of these methods is an important contribution of the MILQ and E-MILQ studies [[15], [16], [17], [18], [19]].

An additional feature of the E-MILQ and MILQ studies is the measurement of milk volume over 14-day periods using the stable isotope dilution dose-to-mother method endorsed by the International Atomic Energy Agency [20,21]. During each assessment window, deuterated water (30 g, 99.8%) was administered to the mothers, and saliva samples were collected from both mothers and infants at baseline and on 1, 2, 3, 4, 13, and 14 d after dosing. The abundance of deuterium in the saliva samples was analyzed by Fourier transformed infrared spectroscopy at St. John’s National Academy of Health Sciences, Bangalore, India. Milk volume intake was calculated from the water flux from mother to infant in a 2-compartment model during the saliva sampling period [21]. In Denmark, volume intake was estimated by 24-h before- and after-feed weighing in the MILQ study, but the deuterium method was used in E-MILQ. In Brazil, milk volume was not assessed in E-MILQ. Total intake of each nutrient was calculated as milk nutrient concentration (μg/L, or mg/L or IU/L) × volume (L) for each mother–infant dyad at each time point.

### Criteria for removal of outliers

Lack of, published cut-points for high or low vitamin or mineral concentrations in human milk complicates decisions about removing outliers. Data were inspected visually as a function of age, and the few extreme outliers (generally >4 SD from the age-adjusted mean or negative values) were identified and removed. Given the number of data points, removing statistical outliers made little difference to the median or percentile values. There are minor differences in sample sizes for nutrients due to different assays.

### Statistical methods

Two types of graphs are presented in the subsequent papers: plots showing nutrient concentration by study site, and estimated percentile curves based on the data. Estimated percentile curves were constructed using generalized additive models for location, scale, and shape (GAMLSS) [22] and the *GAMLSS* package (V5.4-20) using age (in days) as the only explanatory variable. The goal was to find the simplest model that would create smooth curves across time while retaining a good fit. Briefly, these models assume that at each age, the distribution of the variable can be modeled by a family of distributions characterized by ≤4 parameters (generally reflecting location, variability, skewness, and kurtosis) and that these parameters, in turn, can be modeled as a smooth function of age. Weightings were applied to each observation so that each site contributed equal weighting across the full age range. Initial model selection was performed using the *lms()* function, and the performance of further *GAMLSS* family distributions was assessed using the Akaike Information Criterion. The inbuilt *pb()* function, based on *P*-splines and local maximum likelihood, was used to fit the mu, sigma, nu, and tau formulas. Where reducing degrees of freedom did not materially change performance, models were simplified accordingly to minimize overfitting. Model checking was performed with normal Q-Q and worm plots, Q-statistics [23], and finally weighted chi-squared tests for each 30-day interval to assess conformity at all ages to the pre-specified fifth, 10th, 25th, 50th, 75th, 90th, and 95th percentiles. Percentiles were extracted by age in days for plotting and by month for reference tables.

For each milk component, the original plan was to compare the mean of the transformed nutrient concentration between sites and exclude any site whose mean was >0.3 SD larger or smaller than the mean of the other 3 sites on the basis of the difference being statistically and biologically meaningful. However, the results were far more heterogeneous than expected, not only from one variable to the next but across time within the same variable, making it impossible to create consistent criteria for site exclusion. We therefore decided to consider heterogeneous results as representative of the broad variability of human milk across the sites. Future analyses of the MILQ data beyond those included in this supplement will explore potential sources of variability in nutrient concentrations, including maternal diet, inflammation, and genetic polymorphisms.

### Postpartum time of sample collection

The original plan was for the final visit (M3) to include ages 6–8.5 mo, or ≤259 d. Due to unavoidable practical constraints, 4 M3 samples were taken between 259 and 272 days in Brazil, and 44 samples were taken between 259 and 333 d in The Gambia. Rather than removing these observations from analysis, ages beyond the intended range were compressed through logarithmic transformation, and results were retained in the data set.

## Participant data

### Flow of participants

**FIGURE 2:**
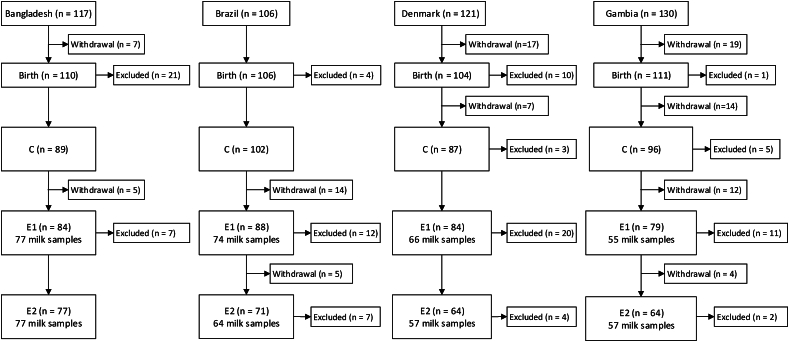
Participant flow diagram for the Early Mothers, Infants, and Lactation Quality (E-MILQ) study. AN, antenatal visit; C, colostrum collection visit; E1, 4–17 d visit; E2, 18–31 d visit.

**FIGURE 3:**
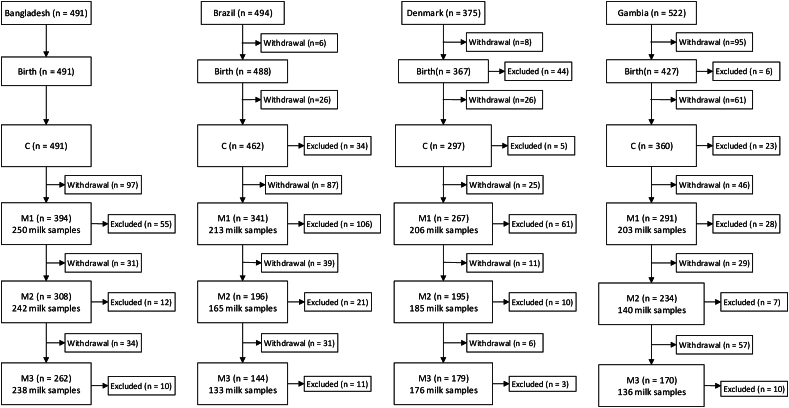
Participant flow diagram for the Mothers, Infants, and Lactation Quality (MILQ) study. AN, antenatal visit; C, colostrum collection visit; M1, 1–3.49 mo visit; M2, 3.5–5.99 mo visit; M3, 6–8.5 mo visit.

FIGURE 2, FIGURE 3 show the flow of participants in the studies. Of 1882 pregnant women who enrolled in the MILQ study across all 4 sites, 1363 (72%), 1016 (54%), and 878 (47%) of mothers were eligible to provide human milk samples at M1, M2, and M3, respectively. Of these, 932, 767, and 707 mothers provided human milk samples. Among mother–infant dyads lost to follow-up, 408, 97, and 88 withdrew before visits M1, M2, and M3, respectively, whereas 361, 50, and 24 were excluded by each of the same visits. The main reasons for exclusion were weight-for-age, height-for-age, or weight-for-length Z-scores <−2 and cessation of exclusive breastfeeding by visit M1. In the E-MILQ study, of 446 mothers who enrolled, 372 (83%) and 316 (71%) were eligible to provide milk samples at E1 and E2. Of these, 294 and 261, respectively, provided milk samples. Among the participants in E-MILQ lost to follow-up, 62 and 9 withdrew before visits E1 and E2, respectively, and 67 and 12 were excluded by the same visits for failing to meet continued inclusion criteria.

### Maternal and infant characteristics

Despite the inclusion and exclusion criteria, maternal and infant characteristics differed among the study sites (TABLE 1, TABLE 2, TABLE 3, TABLE 4) [24]. Literacy was lower in Bangladesh and The Gambia, but still around 90%. At birth, infants weighed less in Bangladesh and The Gambia. Due to our inclusion criteria, none were stunted.

**TABLE 1:** Characteristics of women contributing valid human milk samples to reference value curves, by site (E-MILQ study).

	Bangladesh	Brazil	Denmark	The Gambia	Total	*P*^1^
**n/site** **n/Visit** (d)	77	75	66	63	281	
E1 (4–17)	77	74	66	55	272	
E2 (15–31)	77	64	57	57	255	
**Maternal characteristics**						
Age (y)	25.5 (4.8)	24.9 (5)	31.5 (2.9)	29 (4.7)	27.5 (5.2)	<0.001
BMI (kg/m^2^)^2^	26.2 (3.5)	24.3 (3.2)	22.8 (2.5)	23.5 (4)	24.3 (3.6)	<0.001
Literacy (%)	92.2	98.6	100	88.9	94.9	0.011
Education						<0.001
None	6 (7.8)	0 (0)	0 (0)	11 (17.5)	17 (6.3)	
Primary	23 (29.9)	13 (17.8)	0 (0)	4 (6.4)	40 (14.7)	
Secondary	46 (59.7)	53 (72.6)	0 (0)	42 (66.7)	141 (51.8)	
≥ Graduate	2 (2.6)	7 (9.6)	59 (100)	6 (9.5)	74 (27.2)	
Employment, formal	1 (1.3)	17 (23.3)	51 (85.0)	10 (16.1)		<0.001
**Household size**						
Adults						<0.001
1	1 (1.3)	5 (7.0)	2 (3.3)	1 (1.6)	9 (3.3)	
2	38 (49.4)	44 (62.0)	58 (96.7)	17 (27.0)	157 (57.9)	
3	16 (20.8)	11 (15.5)	0 (0)	7 (11.1)	34 (12.6)	
4	7 (9.1)	9 (12.7)	0 (0)	9 (14.3)	25 (9.2)	
≥5	15 (19.5)	2 (2.8)	0 (0)	29 (46.0)	46 (17.0)	
Children						<0.001
0	28 (36.4)	19 (26.0)	0 (0)	7 (11.1)	54 (19.8)	
1	37 (48.1)	32 (43.8)	38 (63.3)	15 (23.8)	122 (44.7)	
2	12 (15.6)	19 (26.0)	19 (31.7)	15 (23.8)	65 (23.8)	
3	0 (0)	0 (0)	2 (3.3)	8 (12.7)	10 (3.7)	
4	0 (0)	2 (2.7)	0 (0)	8 (12.7)	10 (3.7)	
≥5	0 (0)	1 (1.4)	1 (1.7)	10 (15.9)	12 (4.4)	
**Pregnancy & birth**						
GWG (kg)	6.5 (2.9)	11.2 (6.9)	14.8 (4.4)	6.7 (4.7)	10.6 (6.2)	<0.001
Parity						<0.001
0	25 (32.5)	27 (37.0)	39 (65.0)	22 (34.9)	113 (41.4)	
1	40 (52.0)	28 (38.4)	0 (0)	13 (20.6)	81 (29.7)	
2	10 (13.0)	13 (17.8)	19 (31.7)	11 (17.5)	53 (19.4)	
3	1 (1.3)	1 (1.4)	2 (3.3)	9 (14.3)	13 (4.8)	
4	1 (1.3)	4 (5.5)	0 (0)	6 (9.5)	11 (4.0)	
5+	0 (0)	0 (0)	0 (0)	2 (3.2)	2 (0.7)	
Birth mode						<0.001
C-section	43 (55.8)	7 (9.3)	11 (16.7)	8 (12.9)	69 (24.7)	
Vaginal	34 (44.2)	68 (90.7)	55 (83.3)	54 (87.1)	211 (75.34)	
Birthplace						<0.001
Home	16 (20.8)	0 (0)	5 (7.6)	3 (4.8)	24 (8.6)	
Hospital/clinic	48 (62.3)	75 (100)	61 (92.4)	59 (95.2)	243 (86.8)	
Other	13 (16.9)	0 (0)	0 (0)	0 (0)	13 (4.6)	

NOTE. Data presented as mean (standard deviation), median (interquartile range) or count (%), as appropriate.

Abbreviations: BMI, body mass index; GWG, gestational weight gain.

1 Analysis of variance (ANOVA), Kruskal–Wallis rank sum and Pearson’s chi-squared tests, as appropriate.

2 Prepregnancy in Brazil and Denmark, <2 wk postpartum in Bangladesh and The Gambia.

**TABLE 2:** Characteristics of infants of mothers contributing valid human milk samples to reference value curves by site (E-MILQ study).

	Bangladesh (n = 77)	Brazil (n = 75)	Denmark (n = 66)	The Gambia (n = 63)	Total (n = 281)	
**Birth**						
Weight (g)	2994 (363)	3345 (391)	3511 (344)	3065 (382)	3227 (424)	<0.001
Length (cm)	48.5 (1.3)	48.8 (1.8)	52 (1.8)	48.9 (1.9)	49.5 (2.2)	<0.001
Sex, female (n/%)	40 (52)	40 (53.3)	31 (47)	41 (66.1)	152 (54.3)	0.163
**E1**						
Weight (g)	3153 (350)	3499 (425)	3748 (447)	3419 (527)	3446 (484)	<0.001
Length (cm)	50.1 (1.5)	50.7 (1.7)	52.4 (2.1)	50.3 (1.9)	50.9 (2)	<0.001
Z-scores^1^						
Weight-for-age	−0.8 (0.6)	−0.2 (0.8)	0.3 (0.8)	−0.4 (0.8)	−0.3 (0.8)	<0.001
Length-for-age	−0.6 (0.7)	−0.4 (0.9)	0.5 (1.1)	−0.6 (0.8)	−0.3 (1.0)	<0.001
Weight-for-length	−0.8 (0.7)	−0.1 (0.9)	−0.5 (0.7)	−0.1 (1.0)	−0.4 (0.9)	<0.001
HC (cm)	34.7 (1.0)	35.8 (1.2)	36 (1.4)	35.1 (1.3)	35.4 (1.3)	<0.001
**E2**						
Weight (g)	3696 (381)	4083 (475)	4402 (459)	3969 (560)	4017 (531)	<0.001
Length (cm)	52.2 (1.6)	52.9 (1.8)	55.1 (2)	52.6 (1.9)	53.1 (2.1)	<0.001
Z-scores^1^						
Weight-for-age	−0.7 (0.6)	−0.1 (0.7)	0.4 (0.7)	−0.2 (0.8)	−0.2 (0.8)	<0.001
Length-for-age	−0.6 (0.7)	−0.3 (0.8)	0.7 (1)	−0.4 (0.8)	−0.2 (1)	<0.001
Weight-for-length	−0.5 (0.7)	0.2 (1.1)	−0.5 (0.8)	0 (0.8)	−0.2 (0.9)	<0.001
HC (cm)	35.9 (0.8)	37.1 (1)	37.8 (1.1)	36.3 (1.1)	36.7 (1.3)	<0.001

NOTE. Data presented as mean (standard deviation).

Abbreviation: HC, head circumference.

1 According to World Health Organization Growth Standards [24].

**TABLE 3:** Characteristics of women contributing valid human milk samples to reference value curves, by site (MILQ study).

	Bangladesh	Brazil	Denmark	The Gambia	Total	*P*^1^
**n/site** **n/Visit** (mo)	250	229	206	247	932	
M1 (1.0–3.49)	250	213	206	203	872	
M2 (3.5–5.99)	242	165	185	140	732	
M3 (6.0–8.50)	238	133	176	136	683	
**Maternal characteristics**						
Age (y)	23.9 (4.2)	26.4 (5.7)	31.4 (3.4)	28.3 (4.8)	27.4 (5.4)	<0.001
BMI (kg/m^2^)^2^	25.2 (3.4)	24.1 (3.1)	22.5 (2.4)	23.8 (3.8)	24.0 (3.4)	<0.001
Literacy (%)	91.2	99.1	100	88.2	94.3	<0.001
Education						<0.001
None	25 (10.0)	0 (0)	0 (0)	34 (13.9)	59 (6.4)	
Primary	88 (35.2)	34 (14.9)	0 (0)	22 (9.0)	144 (15.5)	
Secondary	133 (53.2)	171 (75.0)	9 (4.4)	174 (71.0)	487 (52.4)	
≥ Graduate	4 (1.6)	23 (10.0)	197 (95.6)	15 (6.1)	239 (25.7)	
Employment, formal	1 (0.4)	85 (37.3)	166 (80.6)	45 (18.4)	297 (32.0)	<0.001
**Household size**						
Adults						<0.001
1	1 (0.4)	12 (5.3)	3 (1.5)	6 (2.5)	22 (2.4)	
2	102 (40.8)	129 (56.6)	198 (96.1)	63 (25.7)	492 (53.0)	
3	32 (12.8)	39 (17.1)	4 (2.0)	46 (18.8)	121 (13.0)	
4	39 (15.6)	31 (13.6)	0 (0)	32 (13.1)	102 (11.0)	
≥5	76 (30.4)	17 (7.5)	1 (0.5)	98 (40.0)	192 (20.7)	
Children						<0.001
0	87 (34.8)	74 (32.5)	0 (0)	52 (21.2)	213 (22.9)	
1	118 (47.2)	90 (39.5)	145 (70.4)	55 (22.5)	408 (43.9)	
2	32 (12.8)	41 (18.0)	45 (21.8)	38 (15.5)	156 (16.8)	
3	10 (4.0)	14 (6.1)	14 (6.8)	30 (12.2)	68 (7.3)	
4	3 (1.2)	6 (2.6)	2 (1.0)	23 (9.4)	34 (3.7)	
≥5	0 (0)	3 (1.3)	0 (0)	47 (19.2)	50 (5.4)	
**Pregnancy & birth**						
GWG (kg)	7.9 (3.0)	12.1 (5.0)	13.6 (4.3)	7.1 (4.4)	10.4 (5.2)	<0.001
Parity						<0.001
0	101 (40.4)	98 (43.0)	150 (72.8)	97 (39.6)	446 (48.0)	
1	106 (42.4)	73 (32.0)	0 (0)	59 (24.1)	238 (25.6)	
2	30 (12.0)	30 (13.2)	43 (20.9)	45 (18.4)	148 (15.9)	
3	9 (3.6)	13 (5.7)	13 (6.3)	17 (6.9)	52 (5.6)	
4	3 (1.2)	5 (2.2)	0 (0)	18 (7.4)	26 (2.8)	
5+	1 (0.4)	9 (4.0)	0 (0)	9 (3.7)	19 (2.1)	
Birth mode						<0.001
C-section	141 (56.4)	41 (17.5)	12 (5.8)	26 (11.0)	219 (23.8)	
Vaginal	109 (43.6)	189 (82.5)	194 (94.2)	211 (89.0)	703 (76.3)	
Birthplace						<0.001
Home	44 (17.6)	0 (0)	13 (6.3)	8 (3.4)	65 (7.1)	
Hospital/clinic	166 (66.4)	226 (98.7)	193 (93.7)	228 (96.6)	813 (88.3)	
Other	40 (16.0)	3 (1.3)	0 (0)	0 (0)	43 (4.7)	

NOTE. Data presented as mean (standard deviation), median (interquartile range) or count (%), as appropriate.

*Abbreviations:* BMI, body mass index; GWG, gestational weight gain.

1 Analysis of variance (ANOVA), Kruskal–Wallis rank sum and Pearson’s chi-squared tests, as appropriate.

2 Prepregnancy in Brazil and Denmark, <2 wk postpartum in Bangladesh and The Gambia.

**TABLE 4:** Characteristics of infants of mothers contributing valid human milk samples to reference value curves by site (MILQ study).

	Bangladesh (n = 250)	Brazil (n = 229)	Denmark (n = 206)	The Gambia (n = 247)	Total (n = 932)	*P*^1^
**Birth**						
Weight (g)	3102 (379)	3316 (366)	3517 (366)	3112 (399)	3251 (413)	<0.001
Length (cm)	48.2 (1.8)	49.1 (2.1)	51.8 (1.9)	48.9 (2.0)	49.4 (2.4)	<0.001
Sex, female (n/%)	124 (49.6)	115 (50.2)	114 (55.3)	121 (50.0)	474 (51.1)	0.594
**M1** (1–3.49 mo)						
Weight (g)	5498 (760)	5771 (809)	5507 (854)	5549 (937)	5579 (844)	0.002
Length (cm)	58.7 (2.5)	58.7 (2.6)	59.0 (3.0)	58.3 (3.2)	58.7 (2.8)	0.076
Z-scores^1^						
Weight-for-age	−0.49 (0.80)	0.10 (0.88)	0.24 (0.83)	−0.15 (0.82)	−0.09 (0.88)	<0.001
Length-for-age	−0.38 (0.83)	−0.13 (0.86)	0.65 (0.91)	−0.28 (0.92)	−0.05 (0.96)	<0.001
Weight-for-length	−0.21 (0.94)	0.34 (1.02)	−0.31 (0.82)	0.18 (1.0)	−0.01 (0.99)	<0.001
HC (cm)	38.9 (1.4)	39.8 (1.6)	39.4 (1.5)	38.9 (1.6)	39.3 (1.6)	<0.001
EBF	100%	97.8%	100%	99.1%	99.2%	0.02
**M2** (3.5–5.99 mo)						
Weight (g)	7030 (815)	7306 (917)	7203 (930)	6965 (962)	7123 (905)	0.002
Length (cm)	64.8 (2.3)	64.5 (2.6)	65.7 (2.8)	64.4 (2.7)	64.9 (2.6)	<0.001
Z-scores^1^						
Weight-for-age	−0.30 (0.85)	0.14 (0.99)	0.25 (0.86)	−0.25 (0.96)	−0.05 (0.94)	<0.001
Length-for-age	−0.18 (0.85)	−0.12 (0.96)	0.80 (0.95)	−0.15 (0.99)	0.09 (1.02)	<0.001
Weight-for-length	−0.18 (0.97)	0.36 (1.09)	−0.25 (0.90)	−0.13 (1.15)	−0.07 (1.04)	<0.001
HC (cm)	41.4 (1.4)	42.5 (1.5)	42.4 (1.4)	41.7 (1.5)	42.0 (1.5)	<0.001
**M3** (6.0–8.5 mo)						
Weight (g)	7947 (917)	8324 (1058)	8408 (992)	7945 (973)	8137 (997)	<0.001
Length (cm)	68.4 (2.3)	68.4 (2.5)	69.7 (2.6)	68.9 (2.9)	68.9 (2.6)	<0.001
Z-scores^1^						
Weight-for-age	−0.29 (0.91)	0.19 (1.06)	0.42 (0.88)	−0.34 (0.93)	−0.03 (0.99)	<0.001
Length-for-age	−0.34 (0.86)	−0.13 (1.03)	0.64 (0.96)	−0.18 (0.88)	−0.014 (1.0)	<0.001
Weight-for-length	−0.05 (1.01)	0.42 (1.08)	0.20 (0.91)	−0.25 (1.07)	0.06 (1.04)	<0.001
HC (cm)	42.9 (1.4)	44.2 (1.4)	44.4 (1.3)	43.4 (1.4)	43.6 (1.5)	<0.001

NOTE. Data presented as mean (standard deviation).

Abbreviations: EBF, exclusive breastfeeding; HC, head circumference.

1 According to World Health Organization Growth Standards [24].

### Reflections

This introduction to the MILQ and E-MILQ studies is followed by additional articles in this Supplement showing the RVs for macronutrients [25], B vitamins [26], fat-soluble vitamins [27], and minerals [28], and data for milk volume [29]. Supplementary Material shows the percentile values for each nutrient by month postpartum to facilitate the application of the RVs to evaluate milk composition data from other studies. Differences from the composition data used by the National Academy of Medicine (formerly Institute of Medicine) and other groups should not be interpreted as a criticism but rather as an update to the scarce and sometimes inaccurate information available when the nutrient intake recommendations for infants were developed, most of them some 15 y ago. An important point is that the MILQ study was population-based and had a large sample size. Our inclusion and exclusion criteria reduced causes of known variability in milk nutrient concentrations and volume, and eventually, reasons for the variability within this data set will be explored using other study data, including dietary intakes and status biomarkers, and omic variables such as maternal genomics and metabolomics. However, the data should be accepted as revealing the population distribution of RVs in generally well-nourished, healthy women consuming a variety of dietary patterns.

The MILQ study had a number of strengths. Data were collected systematically over time points representative of the comprehensive period through 8.5 mo of lactation. Inclusion criteria were developed to select women who were well-nourished, and milk collection protocols were standardized across 4 study sites that were geographically diverse. Analytical methods were tailored to quantify nutrient concentration in the milk matrix using novel and validated techniques. Milk volume was measured in tandem with nutrient concentration to enable quantification of total nutrient transfer to the infant.

The MILQ study had several limitations. Between sites, data collection methods required occasional modification for cultural sensitivity. For example, in Denmark, milk volume in the MILQ study was measured by test-weighing rather than the deuterium oxide dose-to-mother method. The COVID-19 pandemic, which occurred during the data collection period, resulted in recruitment delays and increased loss-to-follow-up. Although the MILQ study was carried out across 4 geographically diverse sites in Africa, Asia, Europe, and South America, the results are not globally representative.

## Author contributions

The authors’ responsibilities were as follows – LHA, SEM, GK, KM, CM, MI, SS-F, and DH: designed research; SHC, JIL, AMD, DBM, ACF, FK, ADSC, GTS, FN, MH, M.A, BCS, KSJ, DH, and SS-F: conducted research; DH, XT, SS-F, AVK, SD, KSJ, and JMP: analyzed data; DKD, LHA: wrote the paper; LHA: had primary responsibility for the final content and all authors read and approved the final manuscript.

## Data availability

Data described in the manuscript, code book, and analytic code will be made available upon request pending application and approval.

## Funding

This article is published as part of a supplement sponsored by the Gates Foundation (OPP1148405/INV-002300, OPP1061055) and US Department of Agriculture intramural funds (2023-51530-025-00D).

## Conflict of interest

The authors report no conflicts of interest.
